# Chloroquine Is a Zinc Ionophore

**DOI:** 10.1371/journal.pone.0109180

**Published:** 2014-10-01

**Authors:** Jing Xue, Amanda Moyer, Bing Peng, Jinchang Wu, Bethany N. Hannafon, Wei-Qun Ding

**Affiliations:** 1 Department of Pathology, University of Oklahoma Health Sciences Center, Oklahoma City, Oklahoma, United States of America; 2 Department of Radio-Oncology, Nanjing Medical University Affiliated Suzhou Hospital, Suzhou, China; 3 Department of Pharmacology, School of Pharmacy, Xuzhou Medical College, Xuzhou, China; Taipei Medical University, Taiwan

## Abstract

Chloroquine is an established antimalarial agent that has been recently tested in clinical trials for its anticancer activity. The favorable effect of chloroquine appears to be due to its ability to sensitize cancerous cells to chemotherapy, radiation therapy, and induce apoptosis. The present study investigated the interaction of zinc ions with chloroquine in a human ovarian cancer cell line (A2780). Chloroquine enhanced zinc uptake by A2780 cells in a concentration-dependent manner, as assayed using a fluorescent zinc probe. This enhancement was attenuated by TPEN, a high affinity metal-binding compound, indicating the specificity of the zinc uptake. Furthermore, addition of copper or iron ions had no effect on chloroquine-induced zinc uptake. Fluorescent microscopic examination of intracellular zinc distribution demonstrated that free zinc ions are more concentrated in the lysosomes after addition of chloroquine, which is consistent with previous reports showing that chloroquine inhibits lysosome function. The combination of chloroquine with zinc enhanced chloroquine's cytotoxicity and induced apoptosis in A2780 cells. Thus chloroquine is a zinc ionophore, a property that may contribute to chloroquine's anticancer activity.

## Introduction

Chloroquine is an antimalarial drug that has been used in humans for many years [1]. In recent years, Chloroquine has been shown to inhibit autophagy and induce apoptosis in malignant cells and thus has been tested in various experimental model systems [2] and in human clinical trials [3], [4]. Studies have demonstrated that chloroquine sensitizes tumor cells to radiotherapy [5] or chemotherapy [6]–[8]. Therefore, chloroquine may potentially be an effective anticancer drug in clinical oncology. It has also been demonstrated that chloroquine sensitizes breast cancer cells to chemotherapy independent of autophagy inhibition [9], and the potential side effects of chloroquine therapy have also been cautiously discussed [10], [11]. This indicates that a more detailed understanding of chloroquine's anticancer mechanism is required in order to further develop this compound into an effective anticancer agent.

Chloroquine exerts a pleiotropic effect in eukaryotic cells, including an elevation of vacuolar pH when trapped in acidic organelles, such as lysosomes. This increase in pH disrupts lysosomal acidification leading to the impairment of autophagosome fusion and autophagic degradation [12], [13]. At the molecular level, chloroquine has been shown to act synergistically with an Akt inhibitor to induce tumor cell death [14]. However, our understanding of chloroquines' action at the cellular and molecular level in cancer cells is quite limited.

We have previously reported that zinc ions exhibit anticancer activity by altering lysosome membrane permeability [15] and via gene expression regulation [16]. Zinc binding compounds, especially zinc ionophores, are a new group of potential anticancer agents that target zinc to the lysosomes and induce lysosome-mediated apoptosis of cancer cells [17]. In addition, the role of zinc in regulating autophagy has been recently realized [18]. Whereas previous studies have found that metal containing chloroquine complexes may lead to enhanced antimalarial activity [19], its interaction with zinc ions has never been investigated in any biological system. Given the reported anticancer activity of zinc ions and chloroquine and their involvement in lysosomal functions, we sought to investigate whether zinc ions interact with chloroquine and whether this interaction alters chloroquine's anticancer activity. We report that chloroquine is a zinc ionophore, which targets zinc to the lysosomes, and that the combination of zinc and chloroquine enhances their cytotoxicity and induces apoptosis in a human cancer cell model system.

## Materials and Methods

### Materials

The antibody for caspase-3 was from Santa Cruz Biotechnology, Inc. (Santa Cruz, CA). The LC3B-II antibody was from Stressgen (Ann Arbor, MI). The PARP antibody was from Cell Signaling Technology (Danvers, MA). The FluoZin-3 AM probe and LysoTracker probe were purchased from Life Technologies Co. (Carlsbad, CA). CellTiter 96 Aqueous Solution (MTS assay) was from Promega (Madison, WI). Chloroquine diphosphate, zinc chloride, cupric chloride, iron chloride, N,N,N′,N′-Tetrakis(2-pyridylmethyl)ethylenediamine (TPEN), Ca-EDTA, the β-actin antibody and other chemical agents were analytic grade and purchased from Sigma-Aldrich (St. Louis, MO).

### Cell culture

The human ovarian carcinoma cell line A2780 was a kind gift from Dr. Stephen Howell (University of California, San Diego, CA). A2780 cells were cultivated in RPMI 1640 medium supplemented with 10% fetal bovine serum, 100 IU/ml penicillin, and 100 µg/ml streptomycin. Cells were routinely grown in a humidified environment at 37°C, 5% CO_2_, and passaged twice a week. Cells within 20 passages were used in the current study.

### Cell viability assay

Cell viability was analyzed with a modified tetrazolium assay using MTS reagent following the manufacturer's protocol [15]. In brief, A2780 cells were plated in a 96-well tissue culture plate (7.5×10^3^ cells per well) in 100 µl of medium, which ensured a 40–60% cell confluence after 24 hours of growth. The medium was then replaced with 100 µl of fresh medium containing chloroquine and ZnCl_2_ at various concentrations, and the cells were grown for the designated periods. To each well, 20 µl of the MTS solution was added, and cells were incubated at 37°C for 1 hour to allow for color development. The absorbance was recorded at 490 nm and the data were expressed as a percentage of the values obtained from the untreated control cells.

### Intracellular zinc detection

Detection of lysosomes and intracellular free zinc ions was performed using the LysoTracker, and FluoZin-3 probes under fluorescent microscopy following the manufacturer's instructions [20]. A2780 cells were plated in a 12-well plate at a density of 3×10^5^ cells per well. Twenty-four hours after plating, the cells were treated with chloroquine and ZnCl_2_ at the indicated concentrations for 30 min. Following treatment the medium was replaced with fresh medium (RPMI 1640) containing 50 nM LysoTracker or/and 1 µM FluoZin-3. After incubating for another 30 min, the medium was removed and the cells were washed three times with HBSS (Hanks balanced salt solution) and viewed on a Nikon Eclipse TE2000-U microscope. Zinc was detected as a green color (FluoZin-3 AM; excitation 490/20 nm and emission 528/38 nm) and lysosomes as a red color (LysoTracker; excitation 555/28 nm and emission 617/73 nm). Co-localization of zinc ions and lysosomes was determined by merging the green and red images. Fluorescent intensities were quantified using the NIS-Elements AR software (Nikon Instruments).

### Cellular zinc-level measurements

A sensitive and specific form of the high-affinity fluorescent zinc indicator FluoZin-3 was used to determine cellular zinc ion levels [20]. The FluoZin-3 probe was initially prepared in DMSO (5 mM stock) and further diluted in the culture medium prior to addition to the cells. A2780 (1×10^4^ cells per well) were plated in a 96-well plate. Twenty-four hours after plating, the cells were treated with chloroquine for 1 hour in the presence of ZnCl_2_ at the indicated concentrations. FluoZin-3 (1 µM, final concentration) was added and the cells were incubated for another 30 min at 37°C (DMSO concentration in the medium was less than 0.5%). After the incubation, cells were washed twice with fresh medium to remove any dye nonspecifically associated with the cell surface. Additional medium was added and the cells were incubated for another 30 min before fluorescence measurements were made. The fluorescence was measured at 485/535 nm (excitation/emission), using a Wallac 1420 Multilabel Counter (Perkin-Elmer Life Sciences, Boston, MA).

### Western blot analysis

Western blot was performed as described [20], [21]. In brief, cells were lysed with a buffer containing 50 mM Tris (pH 7.4), 50 mM NaCl, 0.5% NP40, 50 mM NaF, 1 mM Na_3_VO_4_, 1 mM phenylmethylsulfonyl fluoride, 25 µg/mL leupeptin, and 25 µg/mL aprotinin. The lysate was sonicated on ice for 6 strokes of 10 seconds each and centrifuged at 15,000×g for 15 min. The supernatants were collected to remove insoluble material. Thirty to forty micrograms of protein from each sample were separated on a 12% SDS-PAGE gel, transferred to a PVDF membrane, and blotted with antibodies against caspase-3, PARP, LC3B-II or β-actin.

### Statistical Analysis

All statistical analysis was performed with Graphpad Prism software (San Diego, CA). One-way ANOVA with Bonferroni analysis was used to determine differences among control and experimental groups, with p<0.05 or p<0.01 as the level of statistical significance. The IC_50_ of chloroquine, in the presence or absence of ZnCl_2_, on cell viability was calculated with a nonlinear regression curve (Sigmoidal dose-response equation).

## Results

### Chloroquine increases zinc uptake in A2780 cells

**Figure 1:**
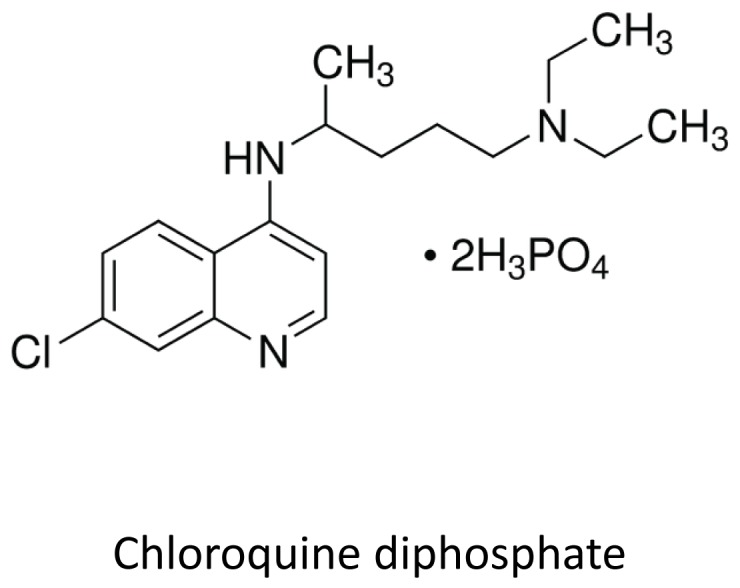
The chemical structure of the chloroquine compound. Chloroquine diphosphate salt was purchased from Sigma-Aldrich (San Louis, MO, C6628).

**Figure 2:**
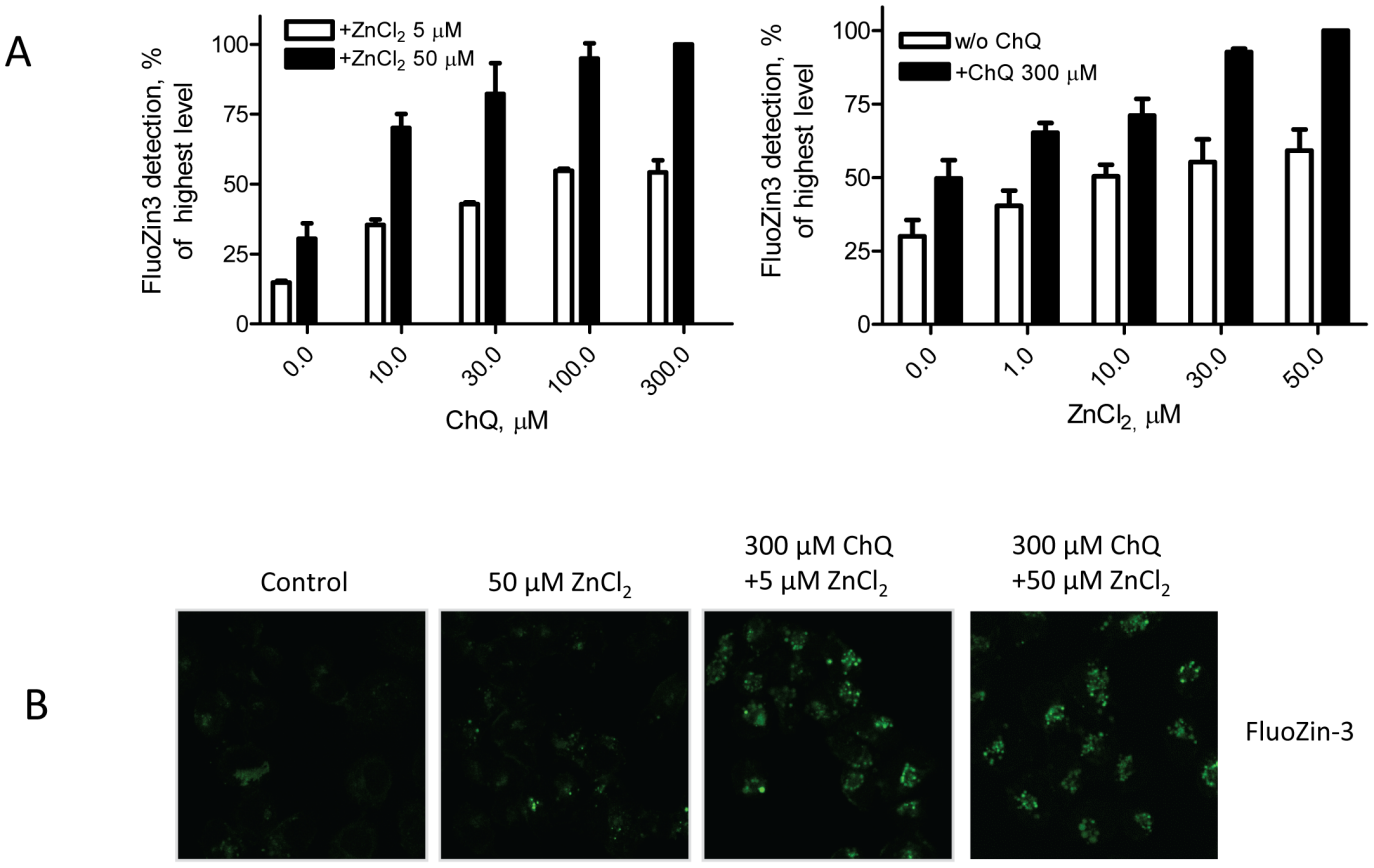
Effects of chloroquine on zinc ion uptake. **A**. A2780 cells were plated in a 96-well plate and treated with chloroquine (ChQ) alone or in combination with ZnCl_2_ at the indicated concentrations for 1 hour. The FluoZin-3 (1 µM) probe was added and the fluorescent signal was measured by excitation at 490/20 nm and emission at 528/38 nm. Data (n = 3, bars, SEM) are presented as a percentage of the highest fluorescence level detected. *, p<0.01, compared to cells without ChQ treatment, using One-way ANOVA followed by Bonferroni analysis. **B**. A2780 cells were plated in a 12-well plate and treated with ChQ and ZnCl_2_ at indicated concentrations for 1 hour. After addition of the FluoZin-3 probe, cells were examined under a fluorescent microscope by excitation at 490/20 nm and emission at 528/38 nm. Shown are representative images from three individual experiments.

**Figure 3:**
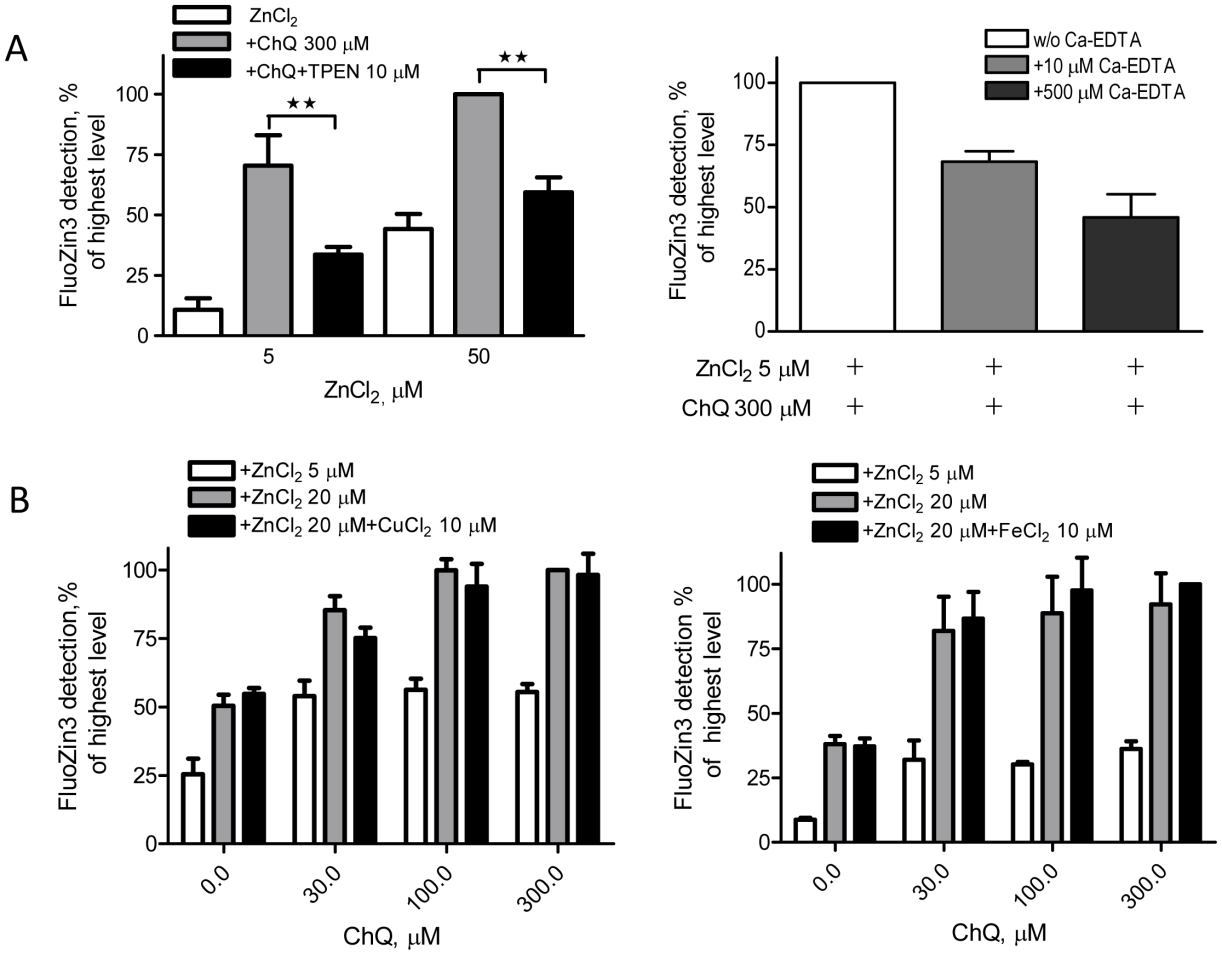
Effects of CuCl_2_, FeCl_2_, TPEN and Ca-EDTA on chloroquine-induced zinc ion uptake. **A**. A2780 cells were plated in a 96-well plate and pretreated with TPEN or Ca-EDTA for 15 min prior to addition of chloroquine (ChQ) and ZnCl_2_ at the indicated concentrations for 1 hour. **B**. A2780 cells were plated in a 96-well plate and treated with ChQ alone or in combination with ZnCl_2_, CuCl_2_, or FeCl_2_ at the indicated concentrations for 1 hour. The FluoZin-3 AM (1 µM) probe was then added and fluorescent signal was recorded by excitation at 490/20 nm and emission at 528/38 nm. Data (n = 3, bars, SEM) are presented as a percentage of the highest fluorescence level detected. *, p<0.01, compared with the fluorescence detected in cells without ChQ treatment, using One-way ANOVA followed by Bonferroni analysis.

To understand whether chloroquine (Figure 1) affects zinc uptake, A2780 cells were treated with 100–300 µM chloroquine in the presence of increased concentrations of zinc chloride for 1 hour. Intracellular basal zinc levels were barely detectable in control cells as assayed using the FluoZin-3 probe and a fluorescent plate reader, as we previously described [15], [17]. Addition of zinc chloride only slightly increased intracellular zinc levels suggesting that internalization of zinc ions is largely limited by the structure of the cell membrane. However, when chloroquine was added to the culture medium intracellular zinc levels were dramatically enhanced (Figure 2A), indicating that chloroquine acts as a zinc ionophore. This enhancement of intracellular zinc levels by chloroquine was clearly dependent on the amount of zinc chloride and chloroquine added (Figure 2A). Fluorescent microscopic examination further demonstrated that zinc uptake is significantly enhanced by chloroquine in this cell model system (Figure 2B). To determine the specificity of the intracellular zinc detection, TPEN, an established membrane permeable high affinity metal-binding compound [22], [23], was added to the cells prior to addition of zinc and chloroquine. As shown in Figure 3A, pretreatment with TPEN significantly attenuated chloroquine-induced intracellular zinc accumulation, indicating that chloroquine specifically brings zinc into the cells. This was further confirmed by addition of iron chloride or copper chloride, which did not affect chloroquine-induced enhancement of intracellular zinc levels (Figure 3B). To be certain that chloroquine does not mobilize zinc ions from intracellular zinc binding molecules, we pretreated the cells with Ca-EDTA, a cell membrane impermeable metal chelator, prior to the addition of chloroquine. As shown in Figure 3A, in the presence of Ca-EDTA chloroquine did not enhance intracellular zinc signaling, further supporting the conclusion that chloroquine is a zinc ionophore.

### Chloroquine targets zinc to the lysosomes

**Figure 4:**
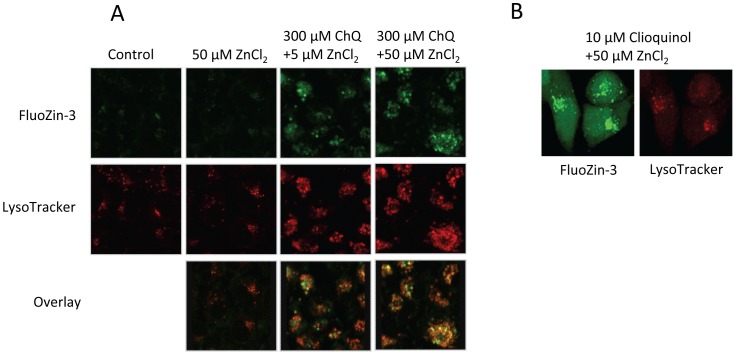
Intracellular zinc ion distribution in A2780 cells after chloroquine treatment. **A**. A2780 cells were plated in a 6-well plate and treated with chloroquine (ChQ) and ZnCl_2_ at the indicated concentrations for 1 hour. After addition of the FluoZin-3 and LysoTracker probes, the cells were examined by confocal microscopy (excitation, 490/20 nm, emission, 528/38 nm for FluoZin-3; 555/28 nm, 617/73 nm for LysoTracker, respectively). Shown are representative images from three individual experiments. **B**. A2780 cells were plated in a 12-well plate and treated with clioquinol (CQ) and ZnCl_2_ at the indicated concentrations for 1 hour. Confocal images were captured as we previously described [17].

In our previous study, we demonstrated that metal ionophores, such as clioquinol, bring zinc ions into the lysosomes of cancer cells leading to lysosome-mediated apoptosis [15]. Because chloroquine is an established lysosomal targeting agent [12], [13], [24], we examined intracellular zinc distribution after treatment of A2780 cells with chloroquine and zinc. As shown in Figure 4, chloroquine induced accumulation of intracellular zinc ions primarily in the lysosomes, as evidenced by co-localization of the fluorescent signals of FluoZin-3 and LysoTracker. Clioquinol, an established zinc ionophore that targets zinc to lysosomes [15], was used as a positive control (Figure 4B). Thus, chloroquine is a zinc ionophore that targets zinc to cellular lysosomes.

### Zinc ions enhance chloroquines' cytotoxicity

**Figure 5:**
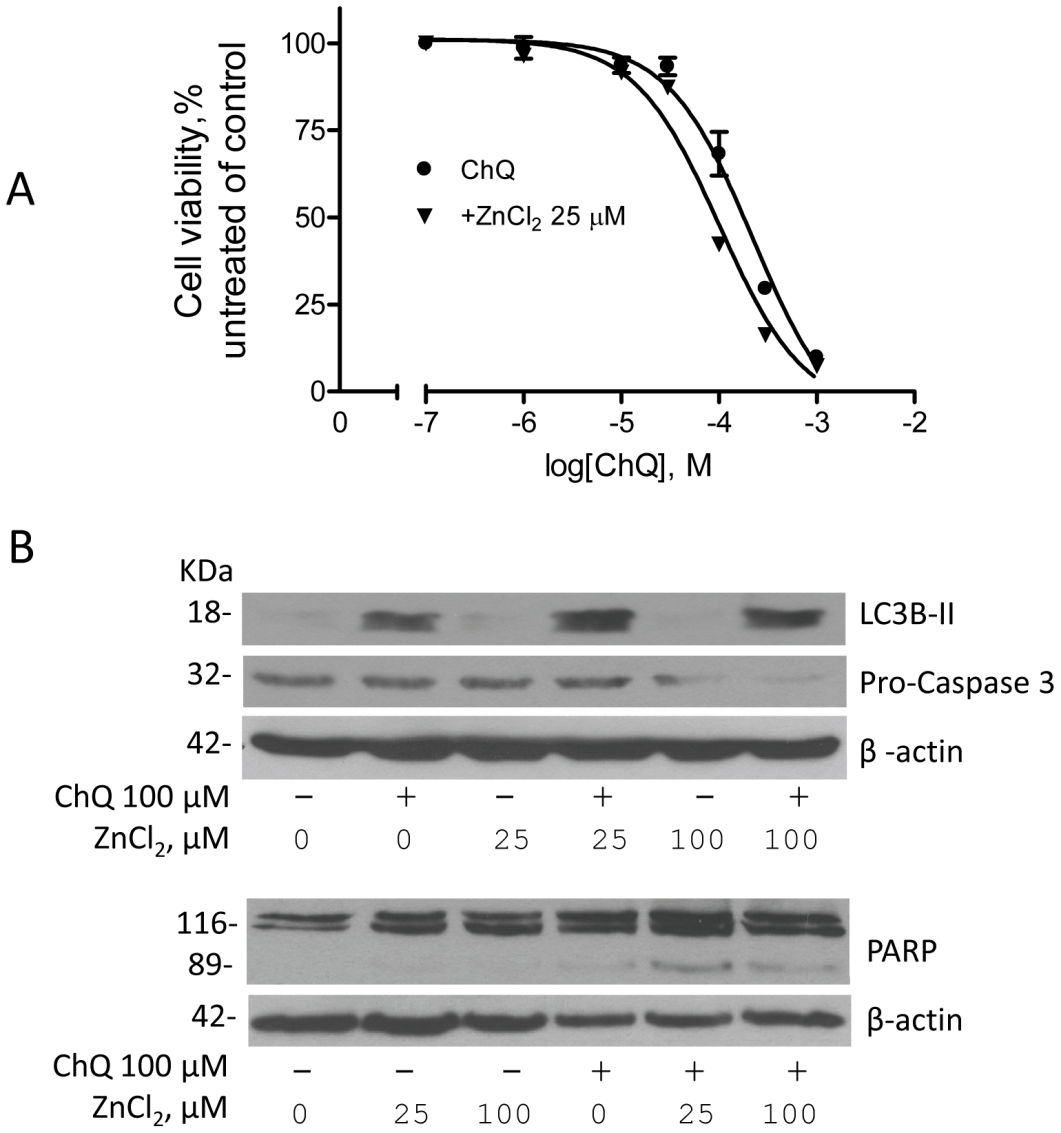
Effects of chloroquine plus zinc on caspase 3 activation and LC3B-II expression. A2780 cells were treated with chloroquine (ChQ) or ZnCl_2_, alone or in combination at the indicated concentrations. Cell lysates were prepared and western blot was performed using primary antibodies against pro-caspase 3, LC3B-II, PARP, and β-actin. Shown are representative blots from three individual experiments.

To determine whether the addition of zinc and chloroquine can kill cancer cells more effectively, A2780 cells were treated with increasing concentrations of chloroquine in the presence of 25 µM zinc chloride for 72 hours. Cell viability analysis indicated that zinc ions significantly enhanced chloroquine's cytotoxicity in this model system (Figure 5A). The IC_50_ of chloroquine, which is typically around 223±10 µM in A2780 cells, was decreased to 101±9 µM in the presence of 25 µM zinc chloride. Furthermore, the combination of chloroquine and zinc treatment significantly enhanced apoptotic cell death as evidenced by caspase-3 activation and PARP cleavage (Figure 5B). Zinc ions also enhanced chloroquine's autophagy inhibitory activity as indicated by LC3B-II detection (Figure 5B).

## Discussion

The novel finding of the present study is that chloroquine is a zinc ionophore, which contributes to our understanding of chloroquine's biological activity in human cancer cells. While chloroquine has been studied in biomedical research [12], [13] and used for the treatment of malaria [1] and other human diseases [25], [26], this activity of the compound has not been previously reported.

The conclusion that chloroquine is a zinc ionophore is based on the detection of significantly elevated intracellular zinc levels when both zinc and chloroquine were added to the cell culture medium. The fluorescent zinc probe used in this study is a well-established intracellular marker for zinc ions and has been validated in previous studies for specific zinc detection [15], [27], [28]. In the current study, the specificity of zinc detection was confirmed by the use of TPEN, a high affinity metal binding compound [22], which blocked chloroquine-induced zinc uptake. Furthermore, in the presence of Ca-EDTA, a membrane impermeable metal chelator, chloroquine did not enhance intracellular zinc levels, indicating that it specifically brings extracellular zinc into the cells to enhance intracellular zinc levels. Interestingly, addition of iron or copper ions did not affect chloroquine-induced zinc uptake, further indicating that chloroquine may primarily act as a zinc ionophore. The affinity of a metal binding compound for different metals can drastically differ [29], therefore whether chloroquine also acts as an ionophore for other metal ions such as copper and iron remains to be determined. The molar concentrations of the metals used in this study were based on our previous reports [15], [23]. While the binding affinity of chloroquine for various metal ions should be further defined, the zinc ionophore activity of this compound provides a new understanding about how chloroquine, in the presence of zinc, may exert its anticancer effects.

We have previously reported that metal binding compounds, such as clioquinol, target zinc to lysosomes thereby inducing apoptosis [15]. The same seems to be true for chloroquine, as evidenced by co-localization of zinc with the lysosome and the induction of apoptosis when cells were treated with the combination of chloroquine and zinc. These data suggest that clioquinol and chloroquine may in part share a similar mechanism in their anticancer action. Both compounds are currently being used in humans for other indications [25], [26], [30] and have proven anticancer activity in experimental model systems [6]–[8], [20]. The fact that chloroquine acts as a zinc ionophore further supports our recent claim that metal ionophores are a new group of anticancer compounds, that merit further exploration [17].

It is interesting to note that the combination of chloroquine and zinc alters LC3B-II protein levels in our model system, indicating that zinc affects chloroquine's inhibitory effect on autophagy. The fact that zinc also enhances chloroquine-induced apoptosis in A2780 cells suggests that inhibition of autophagy and induction of apoptosis by chloroquine is likely a sequential event in this model system. This is consistent with the observation that inhibition of cellular autophagy leads to pro-apoptotic outcomes in human cancer cells [31].

In conclusion, we have identified chloroquine as a zinc ionophore, which reveals new insight into chloroquine's anticancer activity. Since both chloroquine and zinc are considered potential anticancer agents, the combination of the two may be a novel strategy to develop more effective approaches in cancer management.
